# Barriers to ultrasound guidance for central venous access: a survey among Dutch intensivists and anaesthesiologists

**DOI:** 10.1007/s10877-018-00246-z

**Published:** 2019-01-19

**Authors:** Harm J. Scholten, Esther ten Bloemendal, Bente Botter, Hendrikus H. M. Korsten, R. Arthur Bouwman

**Affiliations:** 1 Department of Anaesthesiology, Intensive Care and Pain Medicine, Catharina Hospital, Michelangelolaan 2, 5623 EJ Eindhoven, The Netherlands; 2 Faculty of Electrical Engineering, University of Technology, Eindhoven, The Netherlands; 3 Department of Psychiatry & Medical Psychology, Catharina Hospital, Eindhoven, The Netherlands; 4 Department of Anaesthesiology, Maastricht University Medical Center, Maastricht, The Netherlands

**Keywords:** Ultrasound, Landmark technique, Central venous catherization, Patient safety, Guidelines, Survey

## Abstract

Accumulating evidence shows that ultrasound (US) guidance improves effectiveness and safety of central venous catheter (CVC) placement. Several international guidelines therefore recommend the use of US for placement of CVCs. However, surveys show that the landmark-based technique is still widely used, while the percentage of physicians using US is increasing less than expected. The goal of this study was to investigate current practice for central venous catheterization in anaesthesiology and intensive care in the Netherlands, identify barriers for further implementation of US guidance and to evaluate whether personality traits are associated with the choice of technique. We conducted a web-based national survey, distributed among members of the Dutch societies of anaesthesiology (NVA) and intensive care (NVIC). The survey contained questions regarding physician and hospital characteristics, frequency of US use and reasons for use or non-use, as well as the NEO-FFI-3, a validated, translated questionnaire to characterize personality traits according to the ‘Big Five’ concept. Response rate was 22% (506/2291), of which 400 had also the personality questionnaire complete. Ultrasound guidance was used always or almost always in 68%; barriers for US use were working in a non-academic non-teaching hospital, providing cardiac anaesthesia and more years of physician experience. Reasons for not using US were perceived lack of benefit, increased procedure time, lack of US equipment and fear of loss of landmark technique skills. 13% of respondents had never experienced a complication during CVC placement, and 67% knew of a complication occurring the past year at their department. Ultrasound was thought not to be able to prevent the complication in half of these cases. Of the personality traits, only neuroticism and extraversion showed a minor positive association with US guidance. A majority of anaesthesiologists and intensivists uses US guidance for CVC placement, but a significant proportion of physicians still prefers the landmark technique. Most arguments from respondents against US guidance can be challenged. Personality traits most likely do not play a major role in the acceptance of US guidance for central venous catheterization. A potential intervention to increase US use could be formalizing local hospital policies mandating compliance with US guidance. Future research can perhaps focus on cognitive biases that currently limit more widespread use of US guidance.

**Electronic supplementary material:**

The online version of this article (10.1007/s10877-018-00246-z) contains supplementary material, which is available to authorized users.

## Introduction

Central venous catheters (CVC) are frequently placed in patients who are scheduled for major surgery or admitted to the intensive care unit (ICU). Traditionally, a landmark based technique is used but complications during placement occur in up to 15% of procedures, ranging from relatively mild such as local hematoma or arterial puncture to more serious adverse events such as pneumothorax, retroperitoneal hematoma or cerebral vascular accidents [1]. Ultrasound (US) guidance has consistently shown to not only improve success rate of procedures, but also to decrease complications [2, 3]. The benefits are most prominent for the jugular vein, but also for the subclavian or femoral vein evidence of increasing safety is accumulating [4–7]. Several national societies issued guidelines recommending ultrasound guidance during placement of CVC [8–10], already dating back to 2002 when the National Institute of Clinical Excellence (NICE) recommended US guidance in the United Kingdom. Furthermore, implementation of a local guideline advocating the use of US or the departmental switch from landmark to US results in a reduction of complications [11, 12]. However, previous surveys show that a significant proportion (50–60%) of physicians still prefers the landmark approach in daily practice [13–15]. The most cited reasons for not using ultrasound are lack of benefit, not receiving education in US guidance or lack of US equipment [13–15]. Most of these arguments do not hold anymore as US equipment is ubiquitous available, benefit is proven and training opportunities are universally offered.

Implementations of guidelines can be hampered not only by external barriers but attitudinal aspects also play a role [16]. Furthermore, medical judgment and decisions may be influenced by cognitive errors (biases) or personality traits [17]. Among personality traits, extraversion and neuroticism are known to be negatively correlated with work related safety behaviour [18]. As the use of US guidance can be regarded as a safety enhancing procedure, there may be a relationship between physicians’ personality traits and their adoption of US guidance for vascular access. The most frequently used model to describe personality traits is the ‘Big Five’ concept by Costa and McCrae, allowing rapid characterization of traits into five domains: neuroticism, extraversion, agreeableness, openness to experience and conscientiousness [19].

Hence, the aim of this study was twofold: first, to reassess current practice in central venous access by Dutch anaesthesiologists and intensivists and their attitude regarding US guidance. The second objective was to evaluate the possible association of physicians’ personality traits with clinical practice.

## Methods

An invitation to participate in a web-based survey was sent to all members (including residents) of the Dutch Society of Anaesthesiology (NVA) and to the Dutch Society for Intensive Care (NVIC) using the Qualtrics platform (Qualtrics, Provo, UT, USA). Data was collected from June till September 2017.

The survey was based on previous studies concerning ultrasound guidance in vascular access, with questions regarding personal preferences and attitudes [13–15]. (Supplemental Digital Content 1) For anaesthesiologists, questions regarding US use during regional anaesthesia procedures were added to compare this to CVC insertion practice and assess departmental availability of US equipment.

Personality traits were assessed using the Dutch translation of the revised NEO-FFI-3 [20]. (Supplemental Digital Content 2) The NEO-FFI-3 consists of a 60 item questionnaire assessing the ‘Big Five’ personality traits: neuroticism (N), the tendency to experience emotional distress; extraversion (E), or the tendency to experience positive emotions; conscientiousness (C), the manner of being careful or vigilant; openness to experience (O), or (intellectual) curiosity; and agreeableness (A), being a pro-social person [19]. The items are rated on a five point Likert scale, from totally disagree to totally agree.

The primary outcomes of the study were the frequency of landmark versus US guided CVC placement, and whether physicians’ personality traits were associated with the preference for one or the other technique.

Secondary outcomes were attitudes and perceived barriers regarding the use of US guidance.

### Statistical analysis

The collected data was directly exported from the Qualtrics platform to a SPSS datafile.

Demographical data were described using mean and standard deviation (SD) for numerical variables and numbers or percentages for categorical variables. Unpaired t-test or Mann–Whitney-U test, and Chi^2^ tests were performed to test differences between groups.

To investigate the relationship between personality traits and ultrasound guidance, univariate and multivariate logistic regression was performed. Therefore, the answer regarding usage of US on the five-point scale was transformed to a binary variable; ‘Always’ and ‘Usually’ were combined as ‘frequent use’. ‘Sometimes’, ‘Rarely’ and ‘Never’ were grouped into ‘infrequent use’.

A p value of less than 0.05 was considered statistically significant. All statistical analyses were performed using SPSS Statistics Version 24 (IBM Corp., Armonk, NY, USA).

## Results

A total of 2291 invitations to participate in the survey were sent to consultants and residents in anaesthesiology and intensive care. Overall response rate was 22%, which was divided among 354 anaesthesiologists (23, 6%), 87 residents anaesthesiology (20, 2%) and 156 intensive care physicians (16%) Further details of the respondents are displayed in (Table 1). The median of duration of practice was 11 years. Most respondents (> 80%) work in an academic or teaching hospital setting.

**Table 1:** Demographic variables of respondents

	N	%
Gender		
Male	287	56.7
Years consultant	Median	IQR
	11 (0–38)	5–19
Physician type		
Anesthesiologist	272	53.8
Resident Anesthesiology	86	17.0
Anesthesiologist-Intensivist	72	14.2
Intensivist (other specialty)	66	13.0
Fellow ICU	10	2.0
Cardiac anesthesia		
Yes	51	14.6
Hospital type (consultants)		
Academic	145	34.4
Teaching	193	45.8
Community	83	19.7
Central venous catheters placed annually		
0	9	1.8
1–25 (< 2 monthly)	186	36.8
25–49 (2–4 monthly)	162	32.0
49–100 (1–2 weekly)	84	16.6
> 100 (> 2 weekly)	65	12.8
Peripheral nerve blocks performed weekly		
0	50	13.9
1–5	184	51.0
5–15	96	26.6
> 15	31	8.6

### Central venous catheterization

**Fig. 1:**
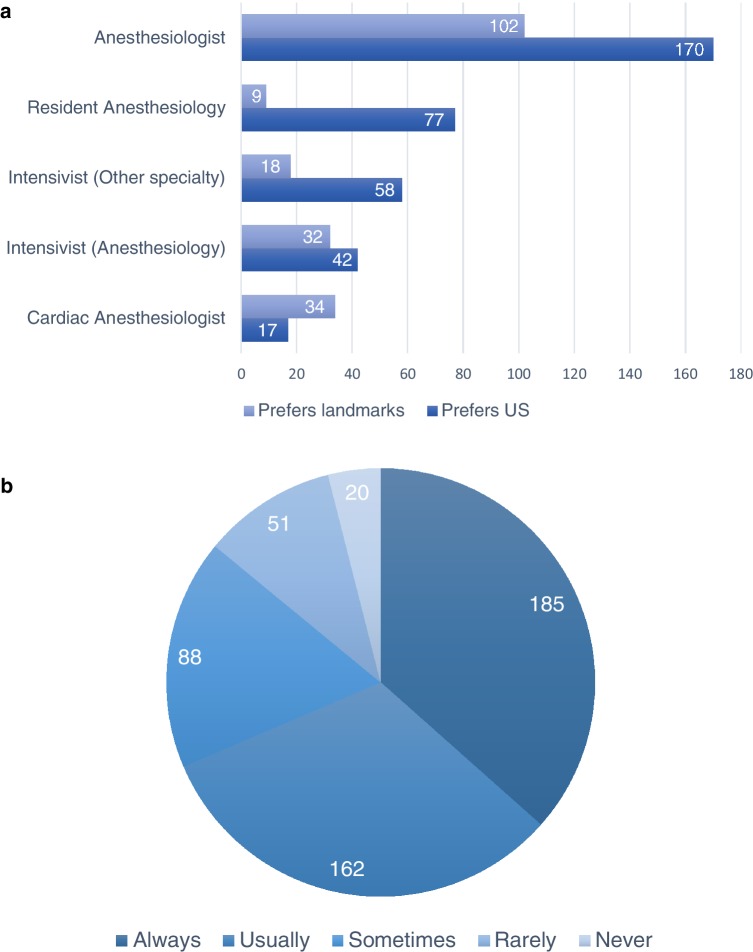
**a** Number of physicians preferring US guidance or landmark, grouped per specialty **b** Total number of physicians preferring US guidance versus landmark

Only nine respondents didn’t perform central venous cannulation. About two-thirds (68.6%) of the respondents always or almost always use US guidance during CVC insertion. (Fig. 1a, b) (Table 2) The most cited reason for not using US guidance was an increase in procedure time (35.8%). Other barriers are the loss of landmark skills (28.6%), lack of US equipment (22.7%) and no perceived benefit of US above the landmark technique 20.9%). On the other hand, higher success rate, less complications and an expected difficult puncture were arguments for the use of US guidance in over half of respondents. When US is used, 13.6% of respondents only locate the vein pre-procedural but the actual puncture is without US guidance. Of the real time users, roughly half prefers in-plane and the other half an out of plane approach. Most physicians experienced at least one complication during CVC placement during their career, of which arterial puncture (69.8%) was the most frequent. During the past year, 67% of respondents recall a complication occurring at their department. In their opinion in half of those cases, US guidance could have prevented the complication. However, in 43% the complication did take place despite using US guidance. An emergency situation where US equipment was not readily available was experienced by 17.2% of the population.

**Table 2:** Description of US guidance for central venous catheter placement

Use of US guidance	n	%
Always	185	36.6
Usually	162	32.0
Sometimes	88	17.4
Rarely	51	10.1
Never	20	4.0
Reasons for not using US guidance (n = 335)		
More time consuming than landmark technique	115	35.8
Loss of skills (landmark technique)	92	28.6
Lack of US equipment	73	22.7
No perceived benefit of US compared to landmarks	67	20.9
Localization (subclavian/femoral vein)	33	10.3
No adequate training/education	25	7.8
Other	36	11.2
Reasons for using US guidance (n = 486)		
Less complications	326	67.1
Higher success rate	286	58.8
Expected difficulty (e.g. obesity)	252	51.9
Education	184	37.9
Coagulation abnormalities	173	35.6
After landmark failed	10	2.1
Other	60	12.3
US technique (n = 481)		
US assisted (anatomic scan, no real time guidance)	69	13.6
Real time out of plane	216	42.7
Real time in plane	196	38.7
Local protocol for CVC insertion		
Yes	349	69.0
Recommending US guidance	264	52.2
No	71	14.0
Did you ever have a complication during CVC insertion		
Never	68	13.1
Arterial punction	363	69.8
Arterial dilation/CVC insertion	70	13.5
Pneumothorax	171	32.9
Hematothorax	26	5.0
Retroperitoneal hematoma	13	2.5
Other	37	7.1
A complication during CVC insertion did occur last year		
Yes	338	67.3
Preventable with US guidance		
Probably	177	52.5
Absolutely not	15	4.5
The complication did occur under US guidance	145	43.0
Experienced emergency situation without US available immediately		
Yes	86	17.2
Would you agree with a national guideline recommending US guidance		
Yes	305	60.3
Yes, but only for the jugular vein	58	11.5
Yes, but only for residents	18	3.6
No	123	24.3

### Personality traits

Complete NEO-FFI-3 inventories were available for 421 respondents. The average scores for males and females are displayed in (Table 3) with the normal references. Female respondents scored higher on the neuroticism scale, but were also more agreeable compared to male respondents. Overall, the respondents scores on all domains were slightly higher than in the general population, except for neuroticism where the scores were just below average. Among specialists, providing cardiac anesthesia was also associated with lower scores on the neuroticism scale.

**Table 3:** Personality traits of respondents

	All			Male			Female			p
	Mean	Range	SD	Mean	Range	SD	Mean	Range	SD	
Neuroticism	27.2	12–51	7.0	26.0	12–46	6.6	28.6	13–51	7.2	< 0.001
Extraversion	43.2	19–56	5.5	43.0	25–56	5.3	43.5	19–56	5.7	0.386
Openness	40.8	24–57	5.7	41.1	24–56	5.2	40.5	26–57	6.1	0.291
Agreeableness	43.2	24–55	4.4	42.4	29–55	4.4	44.2	24–54	4.1	< 0.001
Conscientioussness	47.5	28–59	4.8	47.3	30–59	5.1	47.6	28–59	4.4	0.517

### Regression analysis

We found that both the personality traits neuroticism and extraversion were positively associated with the use of US guidance during CVC placement. The correlation coefficients are displayed in (Table 4). Univariate logistic regression showed significant relationships between not frequently using US and working in a non-academic hospital, placing > 100 CVC’s yearly, male gender, more years of experience, being involved in cardiac anaesthesia and not having a departmental protocol requiring US use. After multivariate analysis, only working in a non-teaching community hospital and providing cardiac anaesthesia were significantly associated with not regularly using US. The results of the logistic regression are displayed in Table 5.

**Table 4:** Regression analysis of personality traits with the use of US guidance during CVC placement

	OR	95% CI	p
Neuroticism	1.047	1.008–1.087	0.017
Extraversion	1.094	1.044–1.145	0.000
Openness	0.985	0.947–1.025	0.451
Agreeableness	1.044	0.994–1.096	0.088
Conscientiousness	0.953	0.905–1.004	0.069

**Table 5:** Univariate and multivariate logistic regression

	Univariate				Multivariate			
	OR	95% C.I		p	OR	95% C.I		p
Type of hospital								
Academic				0.00				0.00
Community, teaching	2.38	1.45	3.91	0.00	1.59	0.75	3.39	0.23
Community, non-teaching	4.77	2.64	8.60	0.00	4.99	1.92	12.98	0.00
Gender								
Male	1.83	1.24	2.71	0.00	1.29	0.68	2.44	0.43
Number of CVCs last year								
1–25 (< 2 monthly)				0.00				0.70
25–49 (2–4 monthly)	1.14	0.71	1.83	0.58	1.43	0.38	5.360	0.60
50–100 (1–2 weekly)	0.99	0.55	1.78	0.98	0.90	0.29	3.42	0.88
> 100 (> 2 weekly)	3.07	1.71	5.51	0.00	1.24	0.36	4.32	0.74
Specialty								
Anesthesia	0.76	0.46	1.24	0.27	0.70	0.31	1.57	0.39
Years experience								
Less than 5 years	0.23	0.15	0.36	0.00	0.51	0.20	1.29	0.15
Less than 10 years	0.32	0.22	0.47	0.00	0.97	0.35	2.63	0.94
Less than 15 years	0.40	0.26	0.60	0.00	0.81	0.33	2.00	0.65
Cardiac anesthesia?	3.96	2.11	7.44	0.00	5.89	1.69	20.49	0.01
Department protocol								
Yes	1.01	0.82	1.25	0.93				
US recommended	0.17	0.10	0.28	0.00	0.00	0.22	0.11	0.44

OR for not using US during CVC placement

## Discussion

Although the number of physicians regularly using US has been gradually increasing since the publication of the NICE guidelines in 2002, still 20–50% of CVC’s is placed using the landmark technique [13–15, 21]. We found that roughly one-third of anaesthesiologists and intensivists in the Netherlands still rely on the landmark technique, despite compelling evidence claiming the superiority of US guidance. This is the first study looking at physicians’ personality traits and their influence on medical decision making concerning US guidance for central venous access.

Factors associated with infrequent use of US guidance were: working in a non-teaching hospital, increasing years of experience and providing cardiac anaesthesia. Gender, number of central vascular procedures performed or type of specialization were not correlated after multivariate logistic regression.

Most cited arguments against the use of US were availability of ultrasound equipment, increase in procedure time, lack of perceived benefit, and loss of (landmark) skills. However, most anaesthesiology or critical care departments own multiple US machines [22]. Moreover, peripheral nerve blocks are performed with US guidance in > 95% by respondents in this study. The present survey shows that cardiac anaesthesiologists are the least frequent users of ultrasound for central venous access. This is a surprising finding, as current practice is that an ultrasound machine is permanent in the cardiac operating room.

Regarding the perceived extra procedural time needed for US guidance, actually the average time decreases using US as multiple misguided punctures will be prevented. But even more important, less skin breaks can decrease complications such as catheter related bloodstream infections [12, 23–25].

Some respondents felt the landmark technique should still be taught, for occasions where US equipment would not be immediately available. First, recognizing external landmarks before continuing the procedure under US guidance is possible, even allowing for better anatomical knowledge or identifying anatomical variations [23, 26]. Interestingly, a significant portion of physicians uses US when landmark technique fails, implicating that using US from the start of the procedure may increase its success ratio.

A complication during catheter placement occurred at the department of 67% of respondents the past year, of which 50% believed the complication was probably preventable with US guidance. Remarkably, 43% of those complications occurred despite using US. An explanation could be not using a real time technique, or known dangers of the out of plane technique as the needle tip is not constantly visualized in this approach, increasing the risk of damaging structures not visible in the US screen. Because of the anonymous design of the survey, no conclusions about departmental protocols and occurrence of complications can be made.

We hypothesized that personality traits may explain the preference for US or landmark technique. We used the ‘Big Five’ model, an established and validated method to characterize personality in five basic traits: neuroticism (or emotional instability), extraversion, agreeableness, openness to experience and conscientiousness [19]. We expected physicians with high scores on extraversion and neuroticism to prefer the landmark technique, as those traits are negatively related to safety related work behaviour [18]. Equally, conscientiousness, agreeableness and openness are linked with a tendency to try new things and to be more precise and careful, so we hypothesized these traits to be associated with US guidance. Contradictory to our hypotheses, we found a very small association between both neuroticism and extraversion and the use of US. For other personality traits we found no effect. When looking at the scores for neuroticism, all respondents have a low score on this scale when compared to the general population. From other scientific disciplines, it is known that a certain degree of neuroticism is associated with more risk aversive behaviour. Put it another way, too little neuroticism can maybe cause an physician underestimate risks and therefore be more resilient in accepting US guidance as a risk lowering strategy for CVC placement are probably turning worries or anxiety into positive behaviour.

Individuals who score higher on neuroticism are known to exhibit risk averse behaviour, especially when also scoring high on conscientiousness [27, 28]. Conversely, openness and extraversion are associated with more risk taking. However, in medicine this relationship has not been studied before [29–31].

Our study has several limitations. The overall response rate of 22% is lower than previous surveys. The past years, a trend in declining response rates of web-based surveys of health care professionals is observed [32, 33]. A proven strategy to improve response rates is a follow up reminder after the initial invitation to non-responders [32, 34]. However, during its distribution many physicians had objections against the personality questionnaire and did not finish this part of the survey. Maybe we struck a nerve on this subject, but we decided not to send the reminder. Moreover, we hypothesize echo enthusiast physicians are more likely to complete the survey, so actual use of US guidance may be lower than we found.

We did not differentiate between locations for CVC placement. The evidence is most clear for the jugular vein, but the principle to visualize needle and target is valid for every location. Indeed, only a minority of respondents cited the location as a reason for not using US.

Our study still leaves some questions unanswered. We demonstrated that US guidance for CVC placement is still not adopted universally despite several guidelines advocating its use. Cabana identified barriers to implement guidelines such as lack of awareness, lack of agreement, external barriers (availability of equipment), the same as we found in our study [35]. In psychological literature, such barriers affecting judgment are called cognitive biases, for instance inertia of previous practice or the ‘status quo’ bias [36, 37]. Another bias occurs when adverse events (e.g. catheter related bloodstream infections) are not directly noted or not communicated back, which is interpreted as positive feedback [38]. Likewise, the ‘overconfidence’ bias, or the ‘above average effect’ results from clinicians (and all other human beings) considering themselves to perform better than their peers, and this confidence is misperceived as competence [39, 40]. Those biases are difficult to overcome by just presenting evidence, as facts that challenge existing assumptions are usually ignored by individuals if they conflict with their personal experiences [41]. Also, physicians are less willing to change their practice when confronted by others [40]. To address all barriers, a combination of education, conversation and skills for motivational change is necessary [42]. Future research and interventions aimed at increasing US guidance for CVC insertion should address those cognitive biases. But despite the reluctancy to accept guidelines, hospitals with a departmental protocol for CVC placement that recommend US guidance had a significantly higher proportion of physicians relying on US guidance.. Additionally, a future scenario with an expanded US-only protocol where X-ray is substituted by US for confirmation of correct central line position and ruling out pneumothorax after insertion, could further lower the barriers for US guidance for placement. However, as availability and acquaintance with US equipment in the cardiac ORs does not seem to have a positive effect, the overall effect of a US-only protocol remains to be seen.

In conclusion, the use of ultrasound guidance for placement of CVCs in the Netherlands has slightly increased the past years but a significant proportion of physicians continues using the landmark technique. Personality traits do not seem to play an important role in the acceptance of ultrasound. Indeed, using landmark technique most CVC’s are placed without adverse events. However, the procedure is performed frequently, possible complications are associated with significant morbidity (and even mortality) and a proven strategy to minimize these complications is directly available. As local guidelines show to increase its use, we encourage department leaders to mandate US guidance in central venous access protocols in order to help the removal of the last barriers for universal use of US guidance.

## Electronic supplementary material

Below is the link to the electronic supplementary material.

Supplemental Digital Content 1 (DOCX 19 KB)

Supplemental Digital Content 2 (DOCX 16 KB)
