# Supplementary material for: Barriers to ultrasound guidance for central venous access: a survey among Dutch intensivists and anaesthesiologists
Source: J Clin Monit Comput. 2019 Jan 19;33(6):1023–31. doi: 10.1007/s10877-018-00246-z (PMC6823316; doi:10.1007/s10877-018-00246-z)
Supplement: Supplementary file 1 — Supplemental Digital Content 1 (DOCX 19 KB) [file 10877_2018_246_MOESM1_ESM.docx]

1. What is your function
   1. Intensivist
   2. Anaesthetist
   3. Resident
2. For intensivists: what is your original specialty?
   1. Anaesthesia
   2. Cardiology
   3. Surgery
   4. Internal medicine
   5. Pulmonology
   6. Neurology
3. What type of hospital ?
   1. Tertiary (academic)
   2. Teaching
   3. Community
4. What year did you register as consultant?
5. Do you give anaesthesia for cardiac surgery?
6. What is your gender?
7. How many peripheral nerve blockaded do you perform weekly?
8. 0
9. 1-5
10. 6-10
11. >10
12. Do you use ultrasound guidance during peripheral nerve block?
    1. Always
    2. Most of the time
    3. Sometimes
    4. Rarely
    5. Never
13. When not using US, please indicate your reasons below (multiple answers allowed)
    1. Availability of equipment
    2. No evidence US is superior to other techniques
    3. Not adequately educated
    4. Increased procedure time
    5. Loss of other techniques
    6. Other …
14. How many central venous catheters do you place annualy ?
    1. 0
    2. 1-25 (<2 monthly)
    3. 25-49 (2-4 monthly)
    4. 49-100 (1-2 weekly)
    5. >100 (>2 weekly)
15. Do you use ultrasound guidance during peripheral nerve block?
    1. Always
    2. Most of the time
    3. Sometimes
    4. Rarely
    5. Never
16. When not using US, please indicate your reasons below (multiple answers allowed)
    1. Availability of equipment
    2. No evidence US is superior to other techniques
    3. Not adequately educated
    4. Increased procedure time
    5. Loss of landmark technique
    6. Other …
17. When using US, please indicate your reasons below (multiple answers allowed)
    1. Less complications compared to landmark
    2. Higher success rate
    3. Expected difficult procedure
    4. Coagulation abnormalities
    5. Teaching purposes
    6. Other …
18. When using US, what is your approach
    1. Preprocedural scan
    2. Real time in plane
    3. Real time out of plane
19. Is there a departmental protocol regarding placement of central venous catheters ?
    1. Yes
    2. No
20. If available, does it recommend using US during placement?
    1. Yes
    2. No
21. Would you think of a national guideline recommending US guidance during CVC placement as justified?
    1. Yes
    2. Yes, but only for non-anaesthesiologists
    3. Yes, but only for the jugular vein
    4. No
    5. Not sure
22. Have you ever had a complication during CVC placement yourself? If so, which complication? (multiple answers possible)
    1. No, never
    2. Carotid artery puncture
    3. Dilatation/catheterization of carotid artery
    4. Pneumothorax
    5. Retroperitoneal hematoma
    6. Other, …
23. Heeft uzelf ooit de a. carotis aangeprikt?
    1. Ja
    2. Nee
24. Is er het afgelopen jaar op uw afdeling een complicatie geweest door het prikken van een centrale lijn?
    1. Yes
    2. No
25. If a complication did occur, would it have been prevented by using US?
    1. Probably
    2. Definitely not
    3. The complication did occur despite US guidance
26. Did you experience an emergency situation, where ultrasound equipment was not available in time?
    1. Yes
    2. No
