# Supplementary material for: Barriers to ultrasound guidance for central venous access: a survey among Dutch intensivists and anaesthesiologists
Source: J Clin Monit Comput. 2019 Jan 19;33(6):1023–31. doi: 10.1007/s10877-018-00246-z (PMC6823316; doi:10.1007/s10877-018-00246-z)
Supplement: Supplementary file 2 — Supplemental Digital Content 2 (DOCX 16 KB) [file 10877_2018_246_MOESM2_ESM.docx]

| NEO Five Factor Inventory – 3 (NEO-FFI-3) |
| --- |
| 1 I am no worrier |
| 2 I like to have lots of people around me |
| 3 I enjoy concentrating on daydreaming |
| 4 I try to give help to anyone in need |
| 5 I keep my belongings neat and clean |
| 6 At times I felt bitter or resentful |
| 7 I laugh easily |
| 8 I spend time learning and developing new hobbies |
| 9 At times I flatter so people do what I want |
| 10 I am pretty good at pacing myself to get things done on time |
| 11 When under great stress, I sometimes feel I’m going to pieces |
| 12 I prefer jobs that let me work alone and unbothered |
| 13 I am intrigued by patterns I find in art and nature |
| 14 Some people think I’m selfish and self-centred |
| 15 I often come to situations less well prepared than others |
| 16 I rarely feel lonely or downhearted |
| 17 I renjoy talking to people |
| 18 If young people hear controversial views it will confuse them |
| 19 If someone starts a fight, I am ready to fight back |
| 20 I perform all tasks given to me conscientously |
| 21 I often feel tense and jittery |
| 22 I like to be where the action is |
| 23 Poetry has little or no effect on me |
| 24 I am better and I know it |
| 25 I have a clear set of goals that are important to achieve |
| 26 I sometimes feel completely worthless |
| 27 I try to avoid crowds |
| 28 I find hard to let my mind wander without control or guidance |
| 29 When insulted, I just try to forgive and forget |
| 30 I waste a lot of time before settling down to work |
| 31 I am rarely fearful or anxious |
| 32 I am often bursting with energy |
| 33 I seldom notice moods or feelings from different environments |
| 34 I tend to assume the best about people |
| 35 I work hard to accomplish my goals |
| 36 I am often angry with treatment by others |
| 37 I am a cheerful, high-spirited person |
| 38 I experience a wide range of emotions or feelings |
| 39 Some people think I am cold and calculating |
| 40 When I make a commitment, I can be counted on |
| 41 Too often I feel discouraged when things go wrong |
| 42 I don’t get much pleasure chatting with people |
| 43 Sometimes from poetry or art, I feel a chill or excitement |
| 44 I have no sympathy for beggars |
| 45 At times I am less dependable and reliable than I should be |
| 46 I am seldom sad or depressed |
| 47 I have fast-paced life |
| 48 I have little interest in speculating on the universe or the human condition |
| 49 Generally I try to be thoughtful and considerate |
| 50 I am a productive person, I always get the job done |
| 51 I often feel helpless, wanting others to solve my problems |
| 52 I am a very active person |
| 53 I have a lot of intellectual curiosity |
| 54 If I don’t like people, I let them know |
| 55 I never seem able to get organised |
| 56 I have been so uncomfortable I wanted to hide |
| 57 I am not interested in leading others |
| 58 Often enjoy playing with theories, abstract ideas (4) agree 3 3.6 |
| 59 If necessary, I am willing to manipulate people to get what I want |
| 60 I strive for excellence in everything I do |
